# Red blood cell distribution width in different time-points of peripheral thrombolysis period in acute ischemic stroke is associated with prognosis

**DOI:** 10.18632/aging.204174

**Published:** 2022-07-13

**Authors:** Mingli He, Hongrui Wang, Yi Tang, Bing Cui, Bingchao Xu, Xiaoqin Niu, Yongan Sun, Guanghui Zhang, Xiaobing He, Bei Wang, Bei Xu, Zaipo Li, Yu Zhang, Yibo Wang

**Affiliations:** 1 Department of Neurology, The Affiliated Lianyungang Hospital of Xuzhou Medical University, Jiangsu, China; 2 State Key Laboratory of Cardiovascular Disease, Fuwai Hospital, National Center for Cardiovascular Diseases, Chinese Academy of Medical Sciences and Peking Union Medical College, Beijing, China

**Keywords:** red blood cell distribution width, thrombolysis, acute ischemic stroke, intracerebral hemorrhage, recurrent stroke

## Abstract

The relationship between red blood cell distribution width (RDW) in peripheral thrombolysis period and prognosis is not fully clarified in those who underwent intravenous thrombolysis (IVT) for acute ischemic stroke (AIS). Our study aimed to clarify this issue. A retrospective analysis of about 510 consecutive thrombolysis cases for AIS from January 2015 to March 2019 in a single-center database was done and followed-up for 3 months. We used univariate and multivariable models to evaluate the relationship between RDW levels at various time-points after IVT and the occurrence risk of hemorrhagic transformation (HT) and recurrent stroke, and used COX regression to assess the hazard ratios of outcomes with RDW levels. Elevated risk of HT was found in higher tertiles of RDW (OR = 10.282, 95% confidence interval (CI) 2.841–39.209, *P* < 0.001 in Tp tertile G3; OR = 5.650, 95% CI 1.992–16.025, *P* = 0.001 in T24 tertile G3; OR = 4.308, 95% CI 1.480–12.542, *P* = 0.007 in T48 tertile G3 and OR = 6.384, 95% CI 2.201–18.515, *P* = 0.001 in T72 tertile G3, respectively). Occurrence of recurrent stroke was highest in the RDW tertile G3 (HR = 4.580, 95% CI 2.123–9.883, *P* < 0.001 in Tp tertile G3; HR = 5.731, 95% CI 2.498–13.151, *P* = 0.001 in T24 tertile G3; HR = 3.019, 95% CI 1.969–4.059, *P* = 0.031 in T48 tertile G3; HR = 3.318, 95% CI 1.598–6.890, *P* = 0.001 in T72 tertile G3, respectively). Mean RDW levels ≥13.60 among AIS patients undergoing thrombolysis was associated with higher risk of HT and recurrent stroke.

## INTRODUCTION

Stroke is one of the leading causes of death and disability in the world [1, 2], recurrent stroke makes up almost 25% of stroke annually [3, 4]. Vessel occlusion and insufficient cerebral perfusion contribute to acute ischemic stroke (AIS) [5, 6]. Hemorrhagic transformation (HT) is believed to be a common complication of AIS and occurs in 10–40% of AIS [7–9]. Several studies revealed that HT was related to worsened prognosis [10–12]. The high disability rate, high occurrence of HT and recurrent stroke will be detrimental to the prognosis of AIS patients. Given the large stroke burden, new biological surrogate markers are needed to identify the occurrence of HT and recurrent stroke for AIS patients and choose appropriate treatment, accordingly may improve the prognosis.

The red blood cell distribution width (RDW) indicates red cell size variation [13–15]. The RDW range differs (11.5–14.5%; 11.5–16%) in normal population according to laboratory normalized values [16, 17]. Elevated RDW level indicates abnormal variation of RBC size in the peripheral blood [15], which is predisposed to thrombophilia due to increased or ineffective red blood cells (RBCs) production and excessive fragmentation or destruction of RBC [18, 19].

RDW has been used to differentiate diagnosis of anemia in clinical setting. Recently, RDW has been recognized as a biomarker for vascular diseases [20, 21]. Previous studies have noted that RDW is a potential independent risk factor for predicting cardiovascular and cerebrovascular diseases [22–27]. A recent study has suggested that AIS patients with increased RDW has higher occurrence of HT [12]. High RDW levels may be can predict independently the occurrence of HT for AIS patients after thrombolysis [24]. However, previous studies on the associations between RDW levels and HT and stroke recurrence in AIS patients treated with IVT were mostly limited to one phase point not to fully explain the full spectrum characteristics of perioperative thrombolysis. Thus, this study aimed to investigate the relationship between RDW levels in the whole peripheral thrombolysis period and prognosis, as well as the temporal changes in post thrombolytic RDW values and their impact on stroke prognosis.

## METHODS

### Selection of patients

**Figure 1:**
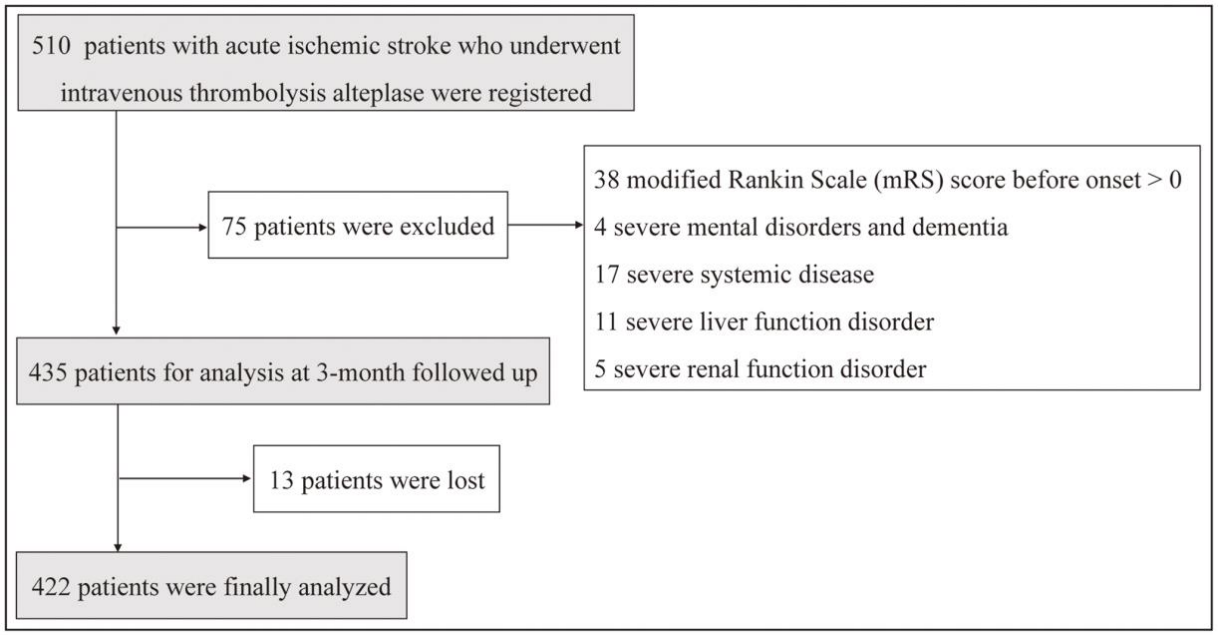
Study flow chart showing the number of patients included in the final analysis.

This was an observational, retrospective, single-center study to determine RDW of peripheral thrombolysis period in AIS is related to prognosis. The current study constituted 510 consecutive IVT-treated AIS patients were admitted to the Department of Neurology of the First People’s Hospital of Lianyungang, Xuzhou Medical University (Jiangsu, China) from January 2015 to March 2019. The participates were inpatient who were definitively diagnosed with AIS and treated with IVT referred to the ‘2014 Chinese guidelines for the diagnosis and treatment of AIS’. Those with severe kidney disease, systemic diseases and kidney disease were excluded from the study population. Finally, 422 AIS patients were eligible for this study. Figure 1 showed the study flow diagram of patients’ enrollment as described previously [5, 28]. Ethical approval for this study was taken from the ethic committee of the First People’s Hospital of Lianyungang City. The approval No. of Ethics Committee was KY20190304002. Written informed consent was obtained from each patient or their relatives.

### RDW measurement

For this study, we planned to include RDW measurements from prior IVT to 72 h after IVT. Blood samples were collected prior IVT, 24 h, 48 h and 72 h after IVT, which were mixed with Ethylenediaminetetraacetic acid (EDTA) and analyzed using the Sysmex automated hematology analyzer (Sysmex Corporation, Kobe, Japan). Two parameters (standard deviation (SD) and coefficient variation (CV)) of RDW were used to measure the extent of anisocytosis. RDW was calculated according to the following formula: RDW (CV) = (SD of erythrocyte volume/mean corpuscular volume) × 100 [20].

The RDW measurement of AIS patients treated with thrombolysis was obtained four time-points: RDW-Tp (prior thrombolysis), RDW-T24 (24 h after thrombolysis), RDW-T48 (48 h after thrombolysis) and RDW-T72 (72 h after thrombolysis). The mean values of RDW levels were calculated by the average of several time-points, including RDW-Tp, RDW-T24, RDW-T48 and RDW-T72.

### Patients’ follow-up and outcome assessment

In this study, the occurrence of HT within 7 days and recurrent stroke of all patients during 3 months were recorded. HT, which was defined as any form of hemorrhage that appears on imaging after IVT, was evaluated using computed tomography (CT) and completed the examination using magnetic resonance imaging (MRI) within 7 days after IVT. Recurrent stroke was defined as occurrence of symptomatic stroke, including ischemic stroke and hemorrhagic stroke. Neurological improvement was defined as an mRS score less than or equal to 2 at 3 months after thrombolysis. All-cause death was defined as death caused by stroke, cardiogenic death and other causes.

### Statistical analysis

Continuous variables were expressed as mean ± SD and were compared using a one-way ANOVA and Kruskal-Wallis H test. Categorical variables were expressed as frequency (percentage) and were analyzed using χ^2^ test or the Fishers accurate test. We categorized patients to three groups (G1–G3) according to the mean RDW levels at different time-points of peripheral thrombolysis period, and the lowest tertile G1 was used as the reference, respectively. Multivariate logistic regression was used as assess the odds ratios (ORs) of HT with RDW levels at different time-points of peripheral thrombolysis period. Variables with *P* < 0.1 in univariate analysis (Supplementary Table 1) were included in the multivariable logistic regression analyses. Model 1 was adjusted for age and sex. Model 2 was further adjusted for other confounders. The Kaplan–Meier model was generated to calculate the probability of recurrent stroke and RDW levels at different time points of peripheral thrombolysis period as a function of time. The differences between the Kaplan–Meier curves were tested for significance by the log-rank test. The risk of a future stroke outcomes for AIS patients was assessed by COX regression analysis. The parameters and reasons of this multiparameter model are the same as multivariable logistic regression analysis (Supplementary Table 2). A two-tailed *P* value less than 0.05 was regarded as statistically significant. All statistical analyses were performed using SPSS.26.0 (IBM, Armonk, New York, USA).

## RESULTS

### Baseline characteristics

510 consecutive candidates were recruited for the study in March 2019. A total of 422 patients were included for the final analyses according to our exclusion criteria. Table 1 showed that the baseline characteristics of the cohort. The prevalence of HT patients was 34 of 422 (8.1%), the prevalence of recurrent stroke patients was 46 of 422 (10.9%) in this study. The mean (SD) age of patients with HT was 66.8 (8.1) years and 20 (58.8%) of patients with HT were men. Although the mean (SD) age of recurrent stroke patients was 67.6 (9.1) years and 28 (60.9%) of recurrent stroke patients were men. The mean RDW levels ranged from 11.38% to 16.49% with a median of 13.60%.

**Table 1:** Demographics of included patients grouped by tertiles of RDW in Tp.

**Characteristics**	**RDW tertiles (%) in Tp**			***P***
	**G1 ≤ 12.44 (*N* = 142)**	**12.44 < G2 ≤ 13.88 (*N* = 141)**	**13.88 < G3 (*N* = 139)**	
RDW values (mean (SD) (min-max))	11.74 (0.40) (11.02–12.44)	13.18 (0.42) (12.46–13.88)	14.60 (0.43) (13.89–15.76)	−
Male (*n*, %)	93 (66.0)	92 (64.3)	89 (64.0)	0.944
Age, years (mean (SD))	65.65 (11.43)	63.75 (11.24)	66.24 (10.29)	0.139
BMI, kg/m^2^	24.77 (3.48)	24.54 (2.75)	24.30 (3.10)	0.447
Height, cm	166.94 (7.87)	166.02 (7.44)	166.10 (7.22)	0.528
Vascular risk factors (*n*, %)				
Hypertension	88 (64.2)	82 (57.7)	87 (62.6)	0.639
Diabetes mellitus	26 (18.4)	19 (13.4)	27 (19.4)	0.350
Atrial fibrillation	22 (15.6)	20 (14.1)	26 (18.7)	0.563
Vascular heart disease	2 (1.4)	8 (5.6)	5 (3.6)	0.160
Coronary atherosclerosis	25 (17.7)	14 (9.9)	15 (10.8)	0.097
Smoking	51 (36.2)	55 (38.7)	57 (41.0)	0.708
Drinking	42 (29.8)	42 (29.6)	33 (23.7)	0.440
IAS (*n*, %)				
No stenosis	69 (48.9)	79 (55.6)	56 (40.3)	0.209
Mild stenosis	25 (17.7)	18 (12.7)	27 (19.4)	
Moderate stenosis	16 (11.3)	14 (9.9)	23 (16.5)	
Severe stenosis	31 (22.0)	31 (21.8)	33 (23.7)	
Long-term medication (*n*, %)				
Hypoglycemic	21 (14.9)	12 (8.5)	23 (16.5)	0.106
Lipid-lowering	13 (9.2)	6 (4.2)	5 (3.6)	0.083
Antiplatelet	23 (16.3)	7 (4.9)	6 (4.3)	<0.001
Anticoagulant	3 (2.1)	3 (2.2)	9 (6.3)	0.089
Antihypertensive	61 (43.3)	62 (43.7)	57 (41.0)	0.889
TOAST classification (*n*, %)				
Aortic atherosclerosis	54 (38.3)	56 (39.4)	68 (48.9)	0.310
Arteriolar occlusive	74 (52.5)	77 (54.2)	59 (42.4)	
Cardiogenic	12 (8.5)	9 (6.3)	12 (8.6)	
Other causes and unknown reasons	1 (0.7)	0 (0.0)	0 (0.0)	
NIHSS score	8.87 (5.50)	9.72 (5.79)	8.86 (6.51)	0.275
BP (mmHg)				
SBP adm	155.94 (22.95)	155.82 (24.65)	161.94 (24.84)	0.055
DBP adm	88.59 (12.52)	89.14 (13.62)	92.19 (15.94)	0.072

Abbreviations: BMI: body mass index; BP: blood pressure; DBP adm: diastolic blood pressure-admission; IAS: intracranial arterial stenosis; ICAS: intracranial atherosclerotic stenosis; NIHSS: National Institute of Health Stroke Scale; SBP adm: systolic blood pressure-admission; SD: standard deviation.

### Comparison of RDW values at different time-points of peripheral thrombolysis period among in patients with and without HT

**Figure 2:**
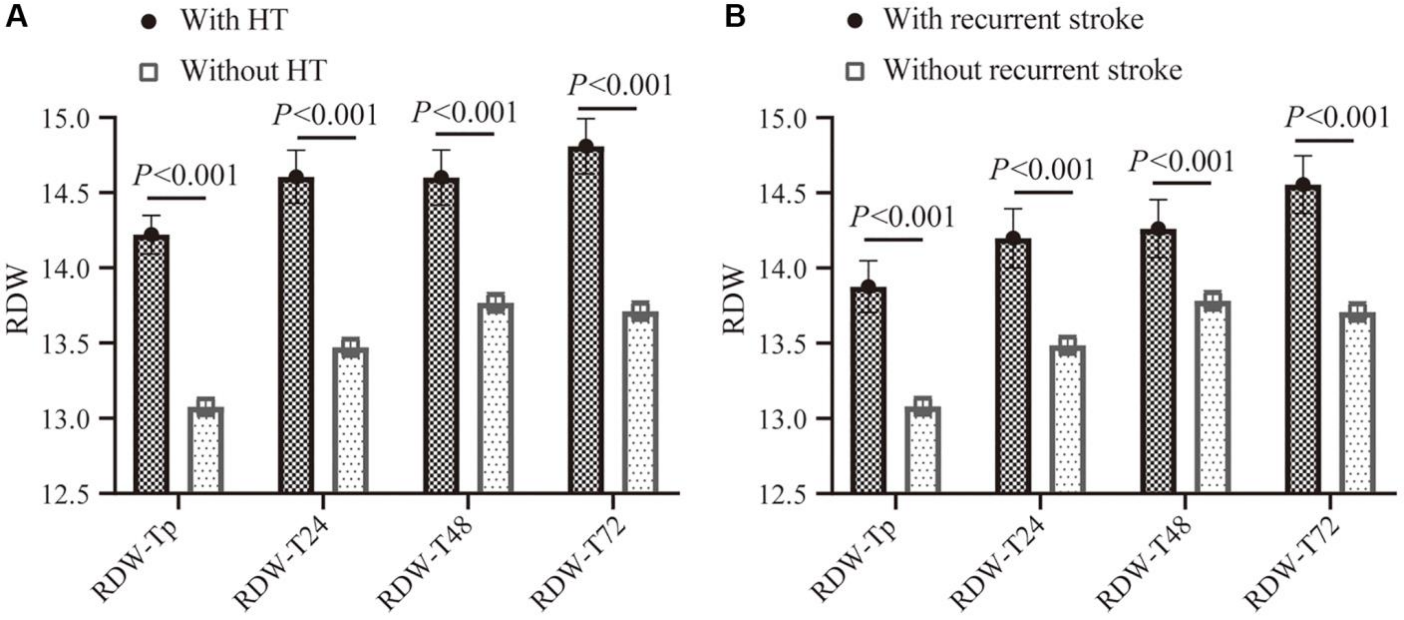
**The RDW profiles with 95% CI in different time-points of peripheral thrombolysis period according to patients with and without HT from onset to 7 days after thrombolysis, or patients with and without recurrent stroke within 3 months.** (**A**) Comparison of RDW levels in different time-points of peripheral thrombolysis period in patients with HT (black) and without HT (gray). (**B**) Comparison of RDW levels in different time-points of peripheral thrombolysis period in patients with recurrent stroke (black) and without recurrent stroke (gray). Abbreviations: RDW: red blood cell distribution width; CI: confidence interval.

The profiles of RDW in AIS patients according to patients with and without hemorrhage on imaging within 7 days after thrombolysis were shown in Figure 2A. The mean RDW was almost lower in patients without HT from prior IVT to 72 h after IVT.

### Relationships between RDW of peripheral thrombolysis period and HT

**Figure 3:**
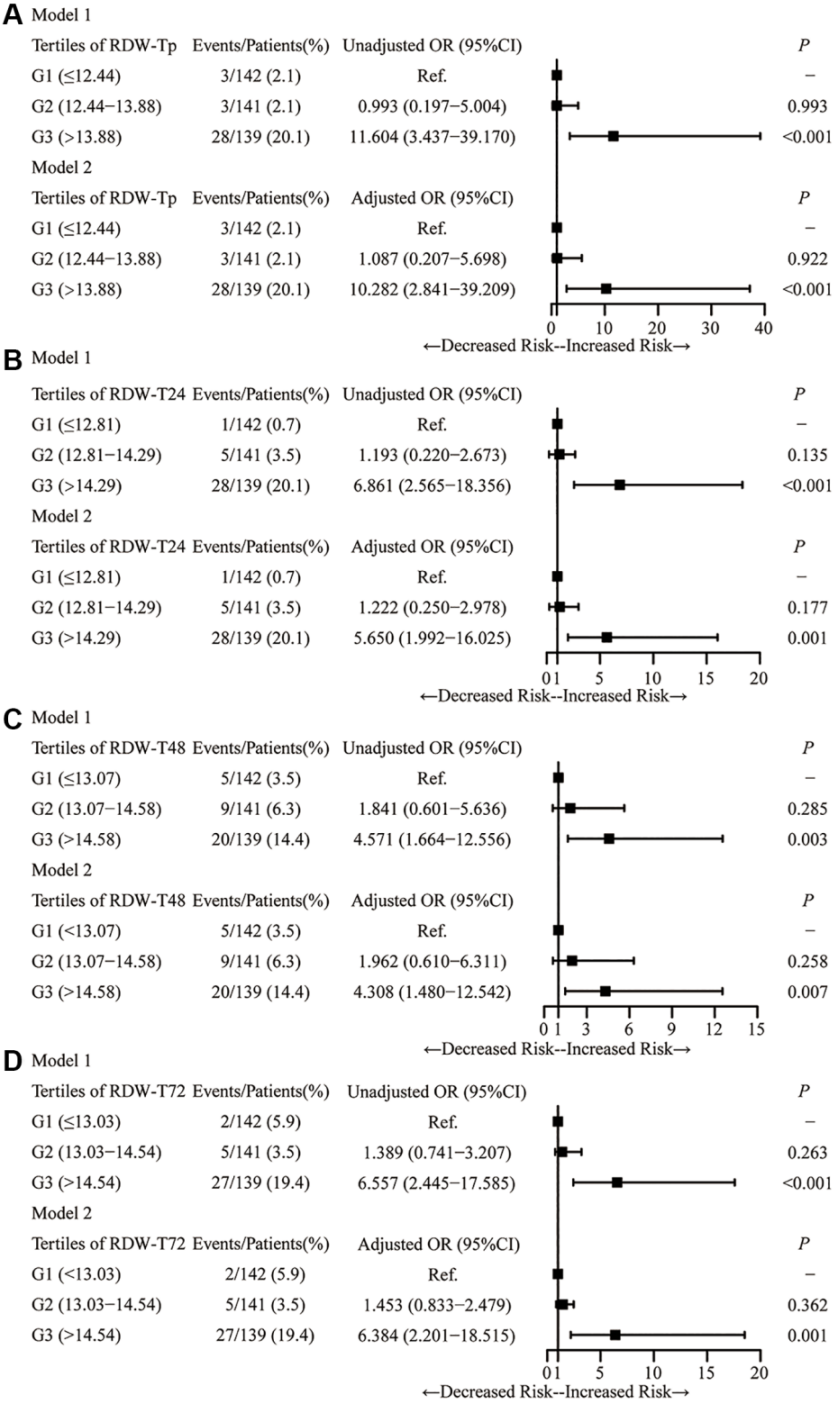
**Logistic analysis demonstrating the relationship between RDW tertiles in the time-points and HT.** Logistic analysis for the association of RDW with HT in Tp (**A**), T24 (**B**), T48 (**C**) and T72 (**D**). Abbreviation: RDW: red blood cell distribution width.

Figure 3 showed the associations between RDW at different time points in prior IVT, 24 h, 48 h and 72 h after IVT and HT after adjusting for multiple variables. Higher risk of HT was found in RDW tertile G3 at different time points in prior IVT, 24 h, 48 h and 72 h after IVT (RDW in prior IVT: OR = 10.282, 95% confidence interval (CI) 2.841–39.209, *P* < 0.001; RDW in 24 h after IVT: OR = 5.650, 95% CI 1.992–16.025, *P* = 0.001; RDW in 48 h after IVT: OR = 4.308, 95% CI 1.480–12.542, *P* = 0.007; RDW in 72 h after IVT: OR = 6.384, 95% CI 2.201–18.515, *P* = 0.001, respectively).

### Comparison of RDW values at different time points of peripheral thrombolysis period among in patients with or without recurrent stroke

The temporal changes of RDW from prior IVT to 72 h after IVT in patients with and without recurrent stroke were shown in Figure 2B. Higher RDW values were observed in patients with recurrent stroke at different time points from prior IVT to 72 h after IVT.

### Associations of RDW tertiles at different time points of peripheral thrombolysis period with recurrent stroke

**Figure 4:**
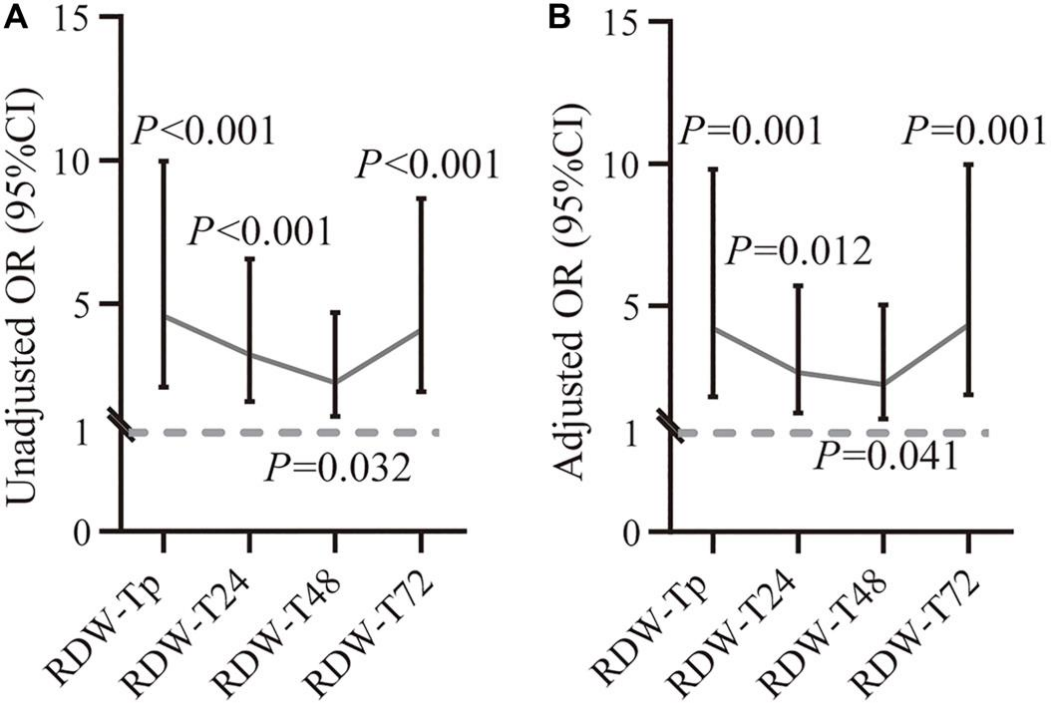
**Associations between RDW at different time points of peripheral thrombolysis period and recurrent stroke.** Correlation between RDW values and recurrent stroke before (**A**) and after (**B**) adjusting variables at different time points from prior thrombolysis to 72 h after thrombolysis. The dashed horizontal lines represent OR values and 95% CI. ^*^P less than 0.05; ^**^P less than 0.01; ^***^P less than 0.001. Abbreviations: CI: confidence interval; OR: odds ratio.

Figure 4 showed the associations between RDW tertiles at different time points of peripheral thrombolysis period and recurrent stroke. Higher risk of recurrent stroke was found in RDW tertile G3 at different time points of peripheral thrombolysis period (RDW in prior IVT: OR = 4.206, 95% CI 1.806–9.796, *P* = 0.001; RDW in 24 h after IVT: OR = 2.651, 95% CI 1.234–5.697, *P* = 0.012; RDW in 48 h after IVT: OR = 2.238, 95% CI 1.016–5.024, *P* = 0.041; RDW in 72 h after IVT: OR = 4.323, 95% CI 1.874–9.970, *P* = 0.001, respectively).

**Figure 5:**
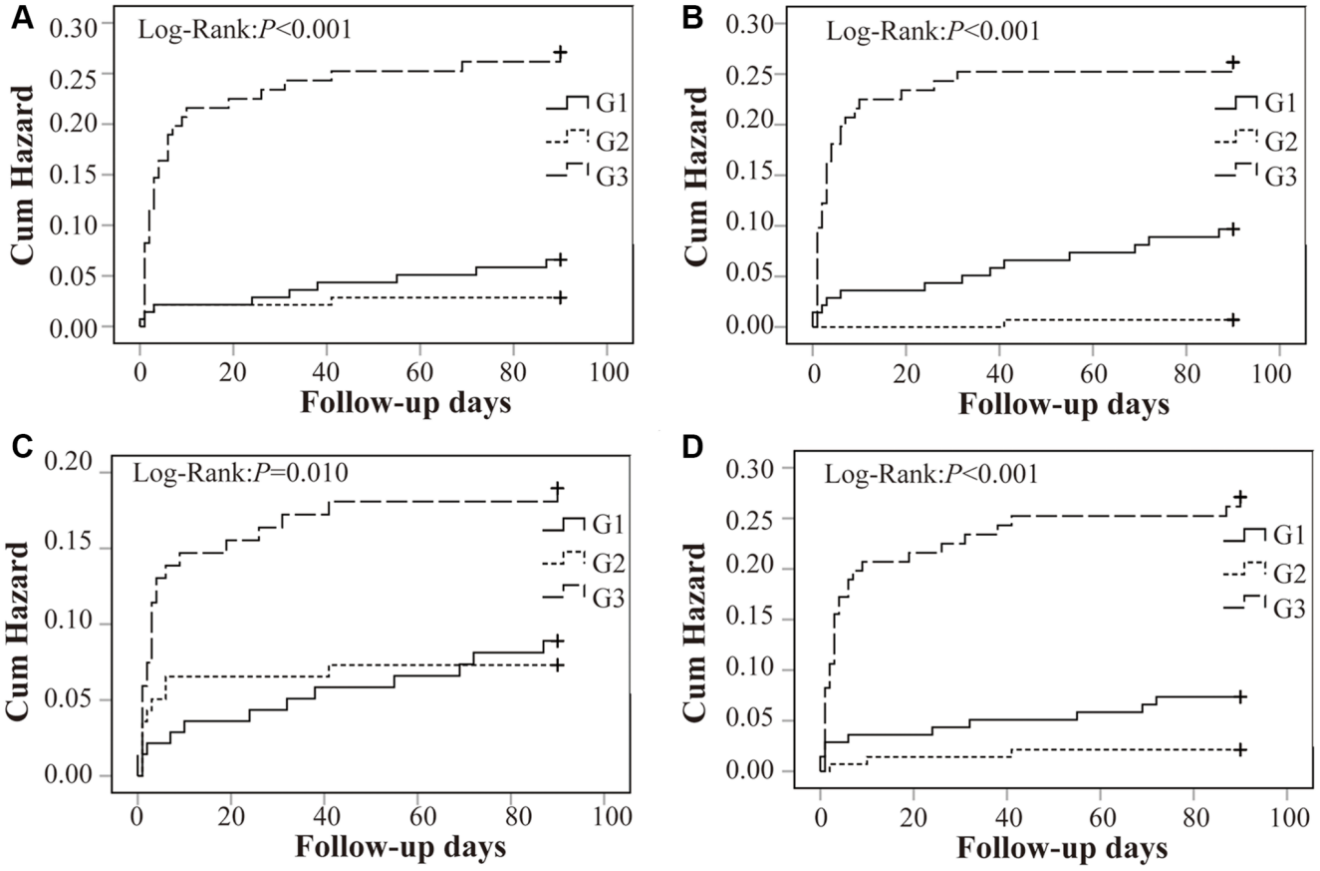
**Kaplan–Meier survival analysis for recurrent stroke within 3 months in relation to RDW tertiles.** RDW was significantly correlated with the increased risk of recurrent stroke in Tp (**A**), T24 (**B**), T48 (**C**) and T72 (**D**). Abbreviation: RDW: red blood cell distribution width.

RDW tertile G3 in prior IVT, 24 h, 48 h and 72 h after IVT were significantly associated with higher risk of recurrent stroke, compared with RDW tertile G1. The Kaplan–Meier curves for recurrent stroke among patients with and without recurrent stroke were shown in Figure 5. Results indicated that the risk of RDW tertile G3 was higher than RDW tertile G1 (RDW in prior IVT: *P* < 0.001; RDW in 24 h after IVT: *P* < 0.001; RDW in 48 h after IVT: *P* = 0.010 and RDW in 72 h after IVT: *P* < 0.001 by log-rank test). Supplementary Figure 1 showed the results of the associations of RDW with recurrent stroke. Occurrence of recurrent stroke was highest in the RDW tertile G3 (RDW in prior IVT: HR = 4.580, 95% CI 2.123–9.883, *P* < 0.001; RDW in 24 h after IVT: HR = 5.731, 95% CI 2.498–13.151, *P* = 0.001; RDW in 48 h after IVT: HR = 3.019, 95% CI 1.969–4.059, *P* = 0.031; RDW in 72 h after IVT: HR = 3.318, 95% CI 1.598–6.890, *P* = 0.001, respectively). However, the differences of between mean RDW levels at different time points of peripheral thrombolysis period and the occurrence of favorable outcome and all-cause death were not observed (Supplementary Figure 2 and Supplementary Table 3).

### Association between mean RDW profiles of peripheral thrombolysis period with prognosis

Supplementary Figure 3 Showed the profiles of mean RDW in AIS patients with and without hemorrhage within 7 days after IVT, or in patients with and without recurrent stroke within 3 months after IVT. Higher mean RDW values were both observed in patients with HT or recurrent stroke in the whole peripheral thrombolysis period (Supplementary Figure 3).

The patients were divided into two groups according to the median (13.60%) of RDW levels. Demographics of included patients grouped by median of mean RDW were shown in Supplementary Table 4. As shown in Table 2, after adjusting for multiple variables including age, sex, diabetes mellitus, lipid-lowering and antiplatelet, higher risk of HT was observed in patients with mean RDW levels ≥13.60 (OR = 8.753, 95% CI 5.130–29.272, *P* < 0.001). After adjusting for multiple variables including age, sex, diabetes mellitus, lipid-lowering, antiplatelet, SBP admission, DBP admission and NIHSS score, mean RDW values ≥13.60 was associated with high risk of recurrent stroke appear (HR = 4.41, 95% CI 2.102–9.235, *P* < 0.001).

**Table 2:** Univariable and multivariable regression analysis of factors affecting HT and recurrent stroke.

**Variables**	**HT**				**Recurrent stroke**			
	**Unadjusted OR (95% CI)**	***P***	**Adjusted OR (95% CI)**	***P***	**Unadjusted OR (95% CI)**	***P***	**Adjusted HR (95% CI)**	***P***
Mean RDW≥13.60	9.75 (5.383–29.554)	<0.001	8.753 (5.130–29.272)	<0.001	4.59 (2.216–9.519)	<0.001	4.41 (2.102–9.235)	<0.001

HT: Adjusted variables, including Age, Sex, Lipid-lowering and Antiplatelet. Recurrent stroke: Adjusted variables, including Age, Sex, Diabetes mellitus, Lipid-lowering, Antiplatelet, SBP adm, DBP adm, NIHSS score. Abbreviations: OR: odds ratio; HR: hazard ratio; RDW: red blood cell distribution width; SBP adm: systolic blood pressure-admission; DBP adm: diastolic blood pressure-admission; NIHSS: National Institute of Health Stroke Scale; HT: hemorrhagic transformation.

## DISCUSSION

Our retrospective analysis of AIS patients treated with IVT revealed that higher mean RDW levels from prior IVT to 72 h after IVT were strongly associated with an increased risk of HT and recurrent stroke. In addition, higher risk of HT and recurrent stroke was found in AIS patients with higher RDW levels at several time-points of peripheral thrombolysis period. Our study aimed to investigate the relationship between RDW levels in the whole peripheral thrombolysis period and stroke prognosis, accordingly provide more evidence support for secondary prevention strategies.

Although hypertension, diabetes mellitus, hyperlipidemia, and higher rates of smoking are the important risk factor of stroke [29–31]. However, the incidence and recurrence rate of stroke have been rising for AIS patients undergoing corresponding treatment. Therefore, with improving advances in stroke medicine, there is necessary to know the biomarkers reflecting the condition and prognosis of patients in order to predict the severity of stroke and compliments the clinical diagnosis, thereby helping in guiding doctors in stroke precision medicine [32]. Clinically, increased RDW is related to anemia caused by iron, folic acid or vitamin B12 deficiency [33]. Elevated RDW is associated with impairment of erythropoiesis, which can reflect chronic inflammation and increased oxidative stress levels [34]. Thus, RDW has been studied as an inflammatory marker in peripheral vascular disease and stroke severity [35–38] In addition, previous studies have revealed that RDW is associated with the occurrence and prognosis of AIS, suggesting that RDW played an important role in the progression of AIS, which may be associated with carotid artery occlusion caused by large RBC [26, 39–41].

HT is the most common complication of AIS patients undergoing IVT therapy and RDW levels are the predictor for the occurrence of HT after thrombolysis [12, 24, 42]. The prevalence of HT in our cohort was 34 (8.06%). Previous studies suggested that elevated RDW levels were independently related to the occurrence of HT of AIS patients before and after thrombolysis [12, 24, 43]. However, their results were concentrated in one time point of peripheral thrombolysis period. Interestingly, our present study showed that AIS patients undergoing IVT with lower RDW levels had lower risk of HT appear, either in prior IVT, 24 h, 48 h or 72 h after IVT, which was consistent with some findings. The mechanism of HT was the increased oxidative damage of blood brain barrier led to vascular leakage and result in the occurrence of HT [44, 45].

RDW has been recognized as a potential independent risk factor to predict the occurrence risk of ischemic cardiovascular and cerebrovascular disease [24]. The potential relationship between RDW levels and stroke was first described in 2008 [35]. The study revealed that higher RDW levels was related to the higher risk of stroke, which was confirmed by other studies [15, 26, 36, 38]. Previous review also revealed higher RDW levels were related to worsen prognosis in AIS patients [43]. Our present study revealed that elevated mean RDW levels from prior IVT and 72 h after IVT or elevated RDW levels at several time-points of peripheral thrombolysis period were also significantly related to higher risk of recurrent stroke, which was in accord with the results of previous studies. Therefore, our results seemed to indicate that RDW could be used as a biomarker for assessing the prognosis of patients with AIS.

In addition, our present results also showed the differences of between mean RDW levels at different time points of peripheral thrombolysis period and the occurrence of favorable outcome and all cause death were not found. This was supported by the study of Kavous Shahsavarinia [46]. They found that no significant difference in mRS between those with normal and higher RDW values either in the 36 h, 7 day or in 3-month. The TESPI trial showed that alteplase was beneficial for patients older than 80 years especially if given within 3 h [47]. Another study revealed that Chinese patients with stroke were younger and their vessel and intracranial atherosclerotic diseases were smaller than patients in high-income countries [48]. Therefore, differences in admission criteria and study population may contribute to this phenomenon. Our results demonstrated that RDW levels in the whole peripheral thrombolysis period were the biomarker reflecting the prognosis of AIS patients undergoing IVT, but the intervention strategy of RDW levels was not clarified. Moreover, the Healthy China campaign included an important part of the prevention and treatment of stroke and the study showed that the effective control rate of primary diseases in high-risk population could be improved by the Stroke Screening and Prevention Program and the complications could be reduced by the Stroke Center and Stroke Unit Care Program, accordingly improve the prognosis of stroke patients [49].

The prevalence of recurrent stroke of AIS patients undergoing IVT after 3 months was 10.9% in the study, which was a little higher than that reported previously [48]. Differences in irregular lifestyle, unhealthy diet, lower education and limited income of patients might be one of causes of recurrent stroke [50, 51]. Decreased awareness of stroke guidelines in community physicians, lack of public knowledge of stroke in population and limited medical care in the regions may be another cause of contributing to the current situation [52–54]. Therefore, a prospective study should be better addressed to provide interventional strategy of RDW levels in AIS patients treated with IVT to reduce their stroke risk.

### Limitations

Our study has some limitations. First, population of the study were from a single center with limited data and shorter follow-up period. Second, HT, which was recognized as a most common complication in clinical prognosis, should be further divided into symptomatic and asymptomatic. Third, blood samples from all patients during the follow-up period were not collected due to limited conditions, so we couldn’t provide the change trend of RDW levels during the follow-up period. Finally, RDW levels and coagulate function and inflammation and oxidative stress between patients with successful reperfusion and those who were not successful reperfusion were not detected. Underlying biological mechanisms about RDW associated with prognosis were not remained to be clarified in the study. Therefore, some possible lacks should be considered as limitations, which have to be better addressed with a prospective study to verify our results and provide interventional strategy of RDW levels to reduce stroke risk.

## CONCLUSION

Our study demonstrated that higher mean RDW level from prior IVT to 72 h after IVT was associated with an increased risk of HT and recurrent stroke. Moreover, patients with recurrent stroke had significantly higher RDW levels at several time-points of peripheral thrombolysis period. The conclusion of this study could be helpful to act RDW as a convenient, fast, and effective diagnostic marker to predict the risk of stroke outcomes for AIS patients in routine physical examination. Further evidence should be needed to determine the optimal RDW levels in the whole peripheral thrombolysis period and provide interventional strategy of RDW levels to reduce stroke risk by a prospective study in the future.

## Supplementary Materials

Supplementary Figures

Supplementary Tables
