# Supplementary material for: Red blood cell distribution width in different time-points of peripheral thrombolysis period in acute ischemic stroke is associated with prognosis
Source: Aging (Albany NY). 2022 Jul 13;14(14):5749–67. doi: 10.18632/aging.204174 (PMC9365566; doi:10.18632/aging.204174)
Supplement: Supplementary Figures [file aging-14-204174-s001.pdf]

SUPPLEMENTARY FIGURES

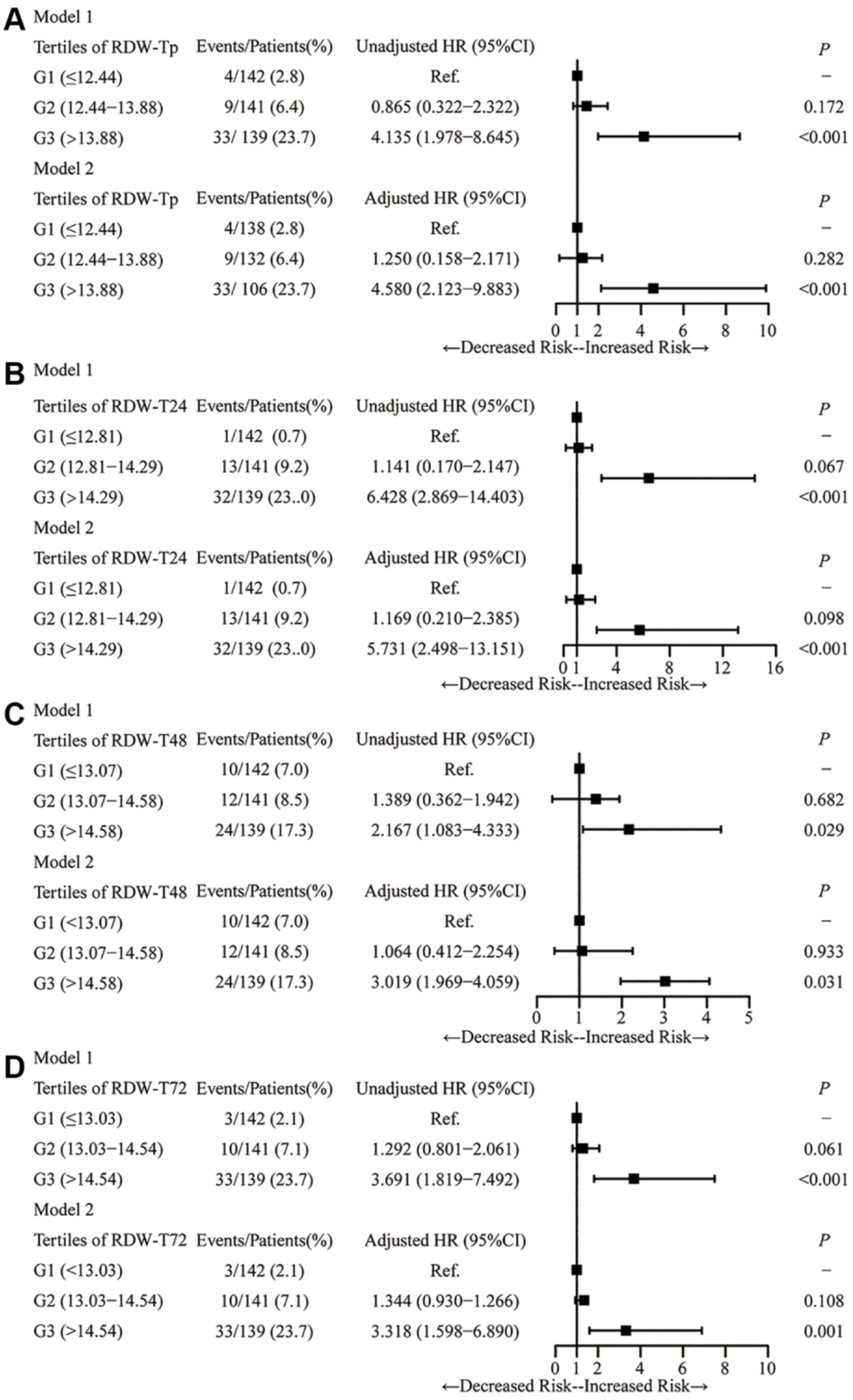

Supplementary Figure 1. Association of RDW levels in different time-points of peripheral thrombolysis period and stroke outcomes. Cox regression analysis demonstrating the relationship between RDW levels in Tp (**A**), T24 (**B**), T48 (**C**), T72 (**D**) and recurrent stroke within 3 months after thrombolysis.

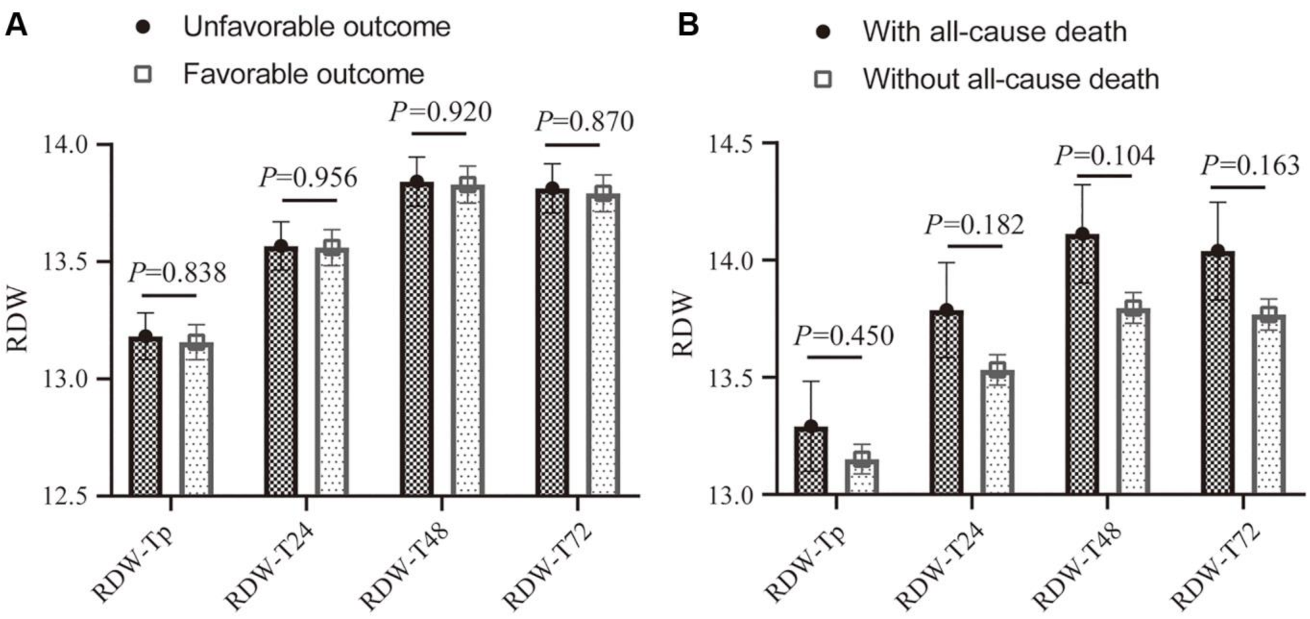

Supplementary Figure 2. The sample RDW profiles with 95% CI in different time-points of peripheral thrombolysis period according to patients with unfavorable outcome and favorable outcome from onset to 7 days after thrombolysis, or patients with all cause death appear and all cause death disappear within 3 months. (**A**) Comparison of RDW levels in different time-points of peripheral thrombolysis period in patients with unfavorable outcome (black) and favorable outcome (gray). (**B**) Comparison of RDW levels in different time-points of peripheral thrombolysis period in patients with all cause death appear (black) and all cause death disappear (gray). Abbreviations: RDW: red blood cell distribution width; CI: confidence interval.

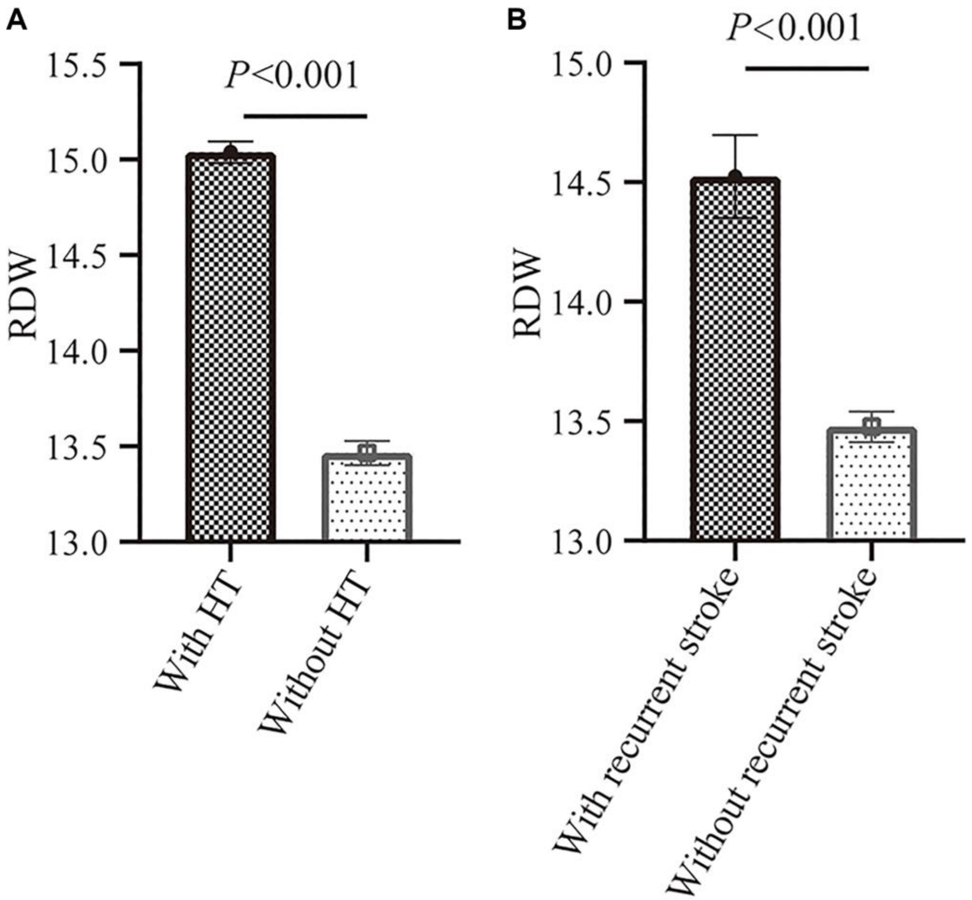

Supplementary Figure 3. The sample mean profiles of RDW with 95% CI according to patients with HT appear and HT disappear from onset to 7 days after thrombolysis, or patients with recurrent stroke and without recurrent stroke within 3 months. (**A**) Comparison of mean RDW levels in different time-points of peripheral thrombolysis period in AIS patients with HT appear (black) and HT disappear (gray). (**B**) Comparison of mean RDW levels in different time-points of peripheral thrombolysis period in AIS patients with recurrent stroke appear (black) and recurrent stroke disappear (gray). Abbreviations: RDW: red blood cell distribution width; CI: confidence interval; HT: hemorrhagic transformation.
