# Supplementary material for: Red blood cell distribution width in different time-points of peripheral thrombolysis period in acute ischemic stroke is associated with prognosis
Source: Aging (Albany NY). 2022 Jul 13;14(14):5749–67. doi: 10.18632/aging.204174 (PMC9365566; doi:10.18632/aging.204174)
Supplement: Supplementary Tables [file aging-14-204174-s002.pdf]

SUPPLEMENTARY TABLES

Supplementary Table 1. Univariate logistic regression to predict HT of AIS patients treated with IVT.

Characteristics	OR (95% CI)	*P*
Male	0.754 (0.369–1.540)	0.438
Age, years	1.015 (0.982–1.049)	0.372
BMI, kg/m^2	1.020 (0.912–1.140)	0.734
Vascular risk factors		
Hypertension	1.194 (0.574–2.482)	0.636
Diabetes mellitus	2.982 (1.401–6.346)	0.005
Atrial fibrillation	1.390 (0.579–3.334)	0.461
Vascular heart disease	0.810 (0.103–6.350)	0.841
Coronary atherosclerosis	0.901 (0.305–2.667)	0.851
Smoking	1.457 (0.721–2.946)	0.294
Drinking	0.933 (0.422–2.064)	0.865
IAS		0.014
Mild stenosis	0.869 (0.232–3.251)	0.834
Moderate stenosis	3.449 (1.288–9.232)	0.014
Severe stenosis	3.076 (1.296–7.296)	0.011
Long-term medication		
Hypoglycemic	3.645 (1.666–7.975)	0.001
Lipid-lowering	0.481 (0.063–3.675)	0.480
Antiplatelet	0.000 (0.00–)	0.998
Anticoagulant	0.810 (0.103–6.350)	0.841
Antihypertensive	1.214 (0.601–2.452)	0.589
TOAST classification		
Arteriolar occlusive	0.274 (0.124–0.604)	0.001
NIHSS score	1.084 (1.026–1.146)	0.004
SBP adm (mmHg)	1.028 (1.013–1.043)	<0.001
DBP adm (mmHg)	1.048 (1.023–1.073)	<0.001

Abbreviations: BMI: body mass index; BP: blood pressure; DBP adm: diastolic blood pressure-admission; IAS: intracranial arterial stenosis; NIHSS: National Institute of Health Stroke Scale; SBP adm: systolic blood pressure-admission; SD: standard deviation.

Supplementary Table 2. Univariate COX regression to predict stroke outcomes of AIS patients treated with IVT.

Characteristics	Primary outcome		Secondary outcome	
	HR (95% CI)	*P*	HR (95% CI)	*P*
Male	0.834 (0.461–1.508)	0.548	0.827 (0.472–1.451)	0.509
Age, years	1.022 (0.994–1.050)	0.127	1.049 (1.019–1.079)	0.001
BMI, kg/m^2	1.017 (0.927–1.115)	0.725	0.944 (0.862–1.032)	0.206
Vascular risk factors				
Hypertension	1.223 (0.667–2.244)	0.515	1.193 (0.672–2.118)	0.548

Diabetes mellitus	2.254 (1.203–4.225)	0.011	1.706 (0.909–3.203)	0.096
Atrial fibrillation	1.110 (0.518–2.380)	0.788	3.125 (1.759–5.551)	<0.001
Vascular heart disease	0.597 (0.082–4.331)	0.610	0.047 (0.000–28.491)	0.350
Coronary atherosclerosis	1.441 (0.672–3.088)	0.348	2.139 (1.120–4.086)	0.021
Smoking	1.483 (0.831–2.644)	0.182	0.861 (0.485–1.529)	0.609
Drinking	1.016 (0.535-1.930)	0.961	0.891 (0.474–1.672)	0.718
IAS		<0.001		<0.001
Mild stenosis	0.579 (0.168–2.001)	0.388	1.135 (0.405–3.184)	0.810
Moderate stenosis	3.948 (1.905–8.182)	<0.001	2.414 (1.001–5.825)	0.050
Severe stenosis	2.119 (1.023–4.390)	0.043	4.670 (2.388–9.132)	<0.001
Long-term medication				
Hypoglycemic	2.775 (1.460–5.273)	0.002	2.357 (1.255–4.424)	0.008
Lipid-lowering	0.745 (0.181–3.075)	0.684	1.187 (0.722–4.573)	0.205
Antiplatelet	0.463 (0.112–1.911)	0.287	1.173 (0.466–2.952)	0.735
Anticoagulant	0.601 (0.083–4.358)	0.614	1.784 (0.556–5.729)	0.331
Antihypertensive	1.244 (0.697–2.218)	0.460	0.859 (0.489–1.506)	0.595
TOAST classification		0.014		<0.001
Arteriolar occlusive	0.399 (0.215–0.742)	0.004	0.168 (0.078–0.360)	<0.001
Cardiogenic	0.165 (0.022–1.209)	0.076	0.890 (0.376–2.110)	0.792
NIHSS score	1.064 (1.013–1.117)	0.013	1.109 (1.067–1.154)	<0.001
SBP adm (mmHg)	1.028 (1.016–1.040)	<0.001	0.998 (0.986–1.009)	0.676
DBP adm (mmHg)	1.040 (1.021–1.059)	<0.001	1.012 (0.993–1.031)	0.230

Abbreviations: BMI: body mass index; BP: blood pressure; DBP adm: diastolic blood pressure-admission; IAS: intracranial arterial stenosis; NIHSS: National Institute of Health Stroke Scale; SBP adm: systolic blood pressure-admission; SD: standard deviation.

Supplementary Table 3. Regression analysis demonstrating the relationship between RDW tertiles in the time-points and neurological improvement and all-cause death.

Tertiles of RDW-Tp	Neurological improvement		All-cause death	
	Unadjusted OR (95% CI)	*P*	Unadjusted HR (95% CI)	*P*
G1 (≤12.44)	–	–	–	–
G2 (12.44–13.88)	1.282 (0.795–2.067)	0.308	0.922 (0.456–1.864)	0.820
G3 (>13.88)	0.894 (0.557–1.433)	0.641	1.280 (0.663–2.470)	0.462
**Tertiles of RDW-T24**	**Unadjusted OR (95% CI)**	***P***	**Unadjusted HR (95% CI)**	***P***
G1 (≤12.81)	–	–	–	–
G2 (12.81–14.29)	1.403 (0.869–2.265)	0.166	0.847 (0.403–1.779)	0.660
G3 (>14.29)	0.894 (0.558–1.432)	0.640	1.600 (0.835–3.066)	0.157
**Tertiles of RDW-T48**	**Unadjusted OR (95% CI)**	***P***	**Unadjusted HR (95% CI)**	***P***
G1 (≤13.07)	–	–	–	–
G2 (13.07–14.58)	1.400 (0.869–2.258)	0.167	0.714 (0.328–1.555)	0.396
G3 (>14.58)	0.975 (0.608–1.562)	0.915	1.756 (0.926–3.330)	0.085

Tertiles of RDW-T72	Unadjusted OR (95% CI)	*P*	Unadjusted HR (95% CI)	*P*
G1 (≤13.03)	–	–	–	–
G2 (13.03–14.54)	1.403 (0.869–2.265)	0.166	0.849 (0.404–1.784)	0.665
G3 (>14.54)	0.894 (0.558–1.432)	0.640	1.608 (0.839–3.082)	0.152

Supplementary Table 4. Demographics of included patients grouped by median of mean RDW.

Variables	Mean RDW<13.60	Mean RDW≥13.60	*P*
Male (*n*, %)	143 (67.5)	131 (62.4)	0.275
Age, years (mean (SD))	64.6 (11.7)	65.8 (10.3)	0.268
BMI, kg/m^2	24.67 (3.22)	24.41 (3.03)	0.380
Height, cm	167.4 (7.8)	166.0 (7.1)	0.045
Vascular risk factors (*n*, %)			
Hypertension	128 (60.4)	129 (61.4)	0.825
Diabetes mellitus	36 (17.0)	36 (17.0)	0.965
Atrial fibrillation	31 (14.6)	37 (17.6)	0.403
Vascular heart disease	5 (2.4)	10 (4.8)	0.182
Coronary atherosclerosis	31 (14.6)	23 (11.0)	0.259
Smoking	80 (37.7)	83 (39.5)	0.706
Drinking	63 (29.7)	54 (25.7)	0.358
IAS (*n*, %)			
No stenosis	103 (48.6)	101 (48.1)	
Mild stenosis	40 (18.9)	30 (14.3)	0.483
Moderate stenosis	23 (10.8)	30 (14.3)	
Severe stenosis	46 (21.7)	49 (23.3)	
Long-term medication (*n*, %)			
Hypoglycemic	26 (12.3)	30 (14.3)	0.541
Lipid-lowering	17 (8.0)	7 (3.3)	0.038
Antiplatelet	27 (12.7)	9 (4.3)	0.002
Anticoagulant	6 (2.9)	9 (4.2)	0.441
Antihypertensive	87 (41.0)	93 (44.3)	0.500
TOAST classification (*n*, %)			
Aortic atherosclerosis	82 (38.7)	96 (45.7)	
Arteriolar occlusive	111 (52.4)	99 (47.1)	0.384
Cardiogenic	18 (8.5)	15 (7.1)	
Other causes and unknown reasons	1 (0.5)	0 (0.0)	
NIHSS score	9.25 (5.92)	9.03 (5.94)	0.695
BP (mmHg)			
SBP adm	157.26 (23.81)	158.50 (24.76)	0.602
DBP adm	89.14 (13.15)	90.79 (15.06)	0.233

Abbreviations: BMI: body mass index; BP: blood pressure; DBP adm: diastolic blood pressure-admission; IAS: intracranial arterial stenosis; ICAS: intracranial atherosclerotic stenosis; NIHSS: National Institute of Health Stroke Scale; SBP adm: systolic blood pressure-admission; SD: standard deviation.
